# Identification of Seven Cell Cycle-Related Genes with Unfavorable Prognosis and Construction of their TF-miRNA-mRNA regulatory network in Breast Cancer

**DOI:** 10.7150/jca.48245

**Published:** 2021-01-01

**Authors:** Zhipeng Hong, Qinglan Wang, Chengye Hong, Meimei Liu, Pengqin Qiu, Rongrong Lin, Xiaolan Lin, Fangfang Chen, Qiuhuang Li, Lingling Liu, Chuan Wang, Debo Chen

**Affiliations:** 1 Department of Breast Surgery, Affiliated Quanzhou First Hospital of Fujian Medical University, Quanzhou, Fujian Province, 362000, P. R. China.; 2 Department of Breast Surgery and General Surgery, The Affiliated Union Hospital of Fujian Medical University, Fuzhou, Fujian Province, 350001, P. R. China.; 3 Breast Cancer Institute, Fujian Medical University, Fuzhou, Fujian Province, 350001, P.R. China.

**Keywords:** bioinformatics analysis, microarray dataset, breast cancer, differentially expressed gene, cell cycle, TF-miRNA-mRNA

## Abstract

Breast cancer (BC), with complex tumorigenesis and progression, remains the most common malignancy in women. We aimed to explore some novel and significant genes with unfavorable prognoses and potential pathways involved in BC initiation and progression via bioinformatics methods. BC tissue-specific microarray datasets of GSE42568, GSE45827 and GSE54002, which included a total of 651 BC tissues and 44 normal breast tissues, were obtained from the Gene Expression Omnibus (GEO) database, and 124 differentially expressed genes (DEGs) were identified between BC tissues and normal breast tissues via R software and an online Venn diagram tool. Database for Annotation, Visualization and Integration Discovery (DAVID) software showed that 65 upregulated DEGs were mainly enriched in the regulation of the cell cycle, and Search Tool for the Retrieval of Interacting Genes (STRING) software identified the 39 closest associated upregulated DEGs in protein-protein interactions (PPIs), which validated the high expression of genes in BC tissues by the Gene Expression Profiling Interactive Analysis (GEPIA) tool. In addition, 36 out of 39 BC patients showed significantly worse outcomes by Kaplan-Meier plotter (KM plotter), and an additional Kyoto Encyclopedia of Genes and Genomes (KEGG) enrichment analysis revealed that seven genes (cyclin E2 (*CCNE2*), cyclin B1 (*CCNB1*), cyclin B2 (*CCNB2*), mitotic checkpoint serine/threonine kinase B (*BUB1B*), dual-specificity protein kinase (*TTK*), cell division cycle 20 (*CDC20*), and pituitary tumor transforming gene 1 (*PTTG1*)) were markedly enriched in the cell cycle pathway. Analysis of the clinicopathological characteristics of hub genes revealed that seven cell cycle-related genes (CCRGs) were significantly highly expressed in four BC subtypes (luminal A, luminal B, HER2-positive and triple-negative (TNBC)), and except for the *CCNE2* gene, high expression levels were significantly associated with tumor pathological grade and stage and metastatic events of BC. Furthermore, genetic mutation analysis indicated that genetic alterations of CCRGs could also significantly affect BC patients' prognosis. A quantitative real-time polymerase chain reaction (qRT-PCR) assay found that the seven CCRGs were significantly differentially expressed in BC cell lines. Integration of published multilevel expression data and a bioinformatics computational approach were used to predict and construct a regulation mechanism: a transcription factor (TF)-microRNA (miRNA)-messenger RNA (mRNA) regulation network. The present work is the first to construct a regulatory network of TF-miRNA-mRNA in BC for CCRGs and provides new insights into the molecular mechanism of BC.

## Introduction

Based on the biennial update of the American Cancer Society on female breast cancer (BC) statistics in the United States, BC is still the most commonly diagnosed malignant disease and the second most common cause of cancer-related death among women after lung cancer 1. In 2018, the number of new diagnoses of female BC cancers worldwide was approximately 2.1 million, leading to approximately 1 in 4 cancer deaths 2. Currently, the main screening methods for BC are limited to traditional mammography, ultrasound, and tumor markers in blood samples, such as carcinoembryonic antigen and carcinoma antigen 15-3 3. In addition, surgical resection, chemotherapy, radiotherapy and hormone therapy are the most commonly chosen treatment strategies in clinical practice. However, there is no doubt that the diagnostic methods and treatment strategies are not precise enough, especially in the current era of precision medicine. Therefore, pursuing more novel and precise therapeutic agents is urgently needed in clinical practice to obtain a more satisfactory prognosis for BC patients.

The tumorigenesis and progression of BC at the molecular level, particularly the genes and pathways involved, are still unknown, and these are helpful for acknowledging tumor behavior and exploring novel targets or strategies for cancer diagnosis or treatment. Excitingly, the rapid expansion of biological microarray analysis greatly promotes the understanding of driver genes and functional pathways in BC by screening DEGs between tumor and normal tissues in the gene expression profiles of tumors 4. Increasing data in various cancers have led to the identification of specific genes or pathways that play important roles in the biology and prognosis of tumors via the use of microarrays and bioinformatics methods 5-8. Although significant expression of DEGs in BC has been reported in some previous studies 9-12, those individual studies provide a relatively limited amount of data regarding carcinogenesis. Hence, integrating multiple sets of BC gene expression data across microarrays to identify significant DEGs via bioinformatics analysis is a promising approach to demonstrate the potential driver genes, associated pathways and interaction networks underlying BC.

In the present study, a cross-tissue gene expression comparison in BC was conducted by integrating three BC gene expression datasets, which contained stroma samples from invasive ductal carcinoma and breast tissues. Differential analysis, enrichment analysis, protein-protein interaction (PPI) network analysis, RNA sequencing expression analysis, survival analysis, clinicopathological characteristics analysis, mutation analysis and quantitative real-time polymerase chain reaction assay were performed to identify hub genes associated with BC and to further construct a transcription factor (TF)-microRNA (miRNA)-messenger RNA (mRNA) regulatory network. While revealing the potential molecular mechanism of BC occurrence, these findings may provide a basis for developing potential therapeutic targets for BC.

## Materials and Methods

### Collection of microarray data information from GEO datasets

The Gene Expression Omnibus (GEO; https://www.ncbi.nlm.nih.gov/geo/) is known as a public functional genomics data repository containing array- and sequence-based data, which allows users to search, browse and download gene expression profiles. After careful review and comparison, we obtained three gene expression profiles of BC and normal breast tissues, namely, GSE42568, GSE45827 and GSE54002. Microarray datasets of GSE42568, GSE45827 and GSE54002 were all processed on the GPL570 platform ([HG-U133_Plus_2] Affymetrix Human Genome U133 Plus 2.0 Array) and contained 104 BC tissues and 17 normal breast tissues, 130 BC tissues and 11 normal breast tissues, and 417 BC tissues and 16 breast tissues, respectively.

### Data processing and differential analysis

We detected and removed batch effects in each dataset (Supplementary Figure S1) and then classified the BC specimens and normal breast specimens in three microarray datasets using R software (version 3.4.4) packages edgeR and limma to identify DEGs. The cutoff criteria were |log_2_FC| >2 and adjusted *p* value <0.05. Next, DEGs in each dataset were exported to an online Venn diagram tool (http://bioinformatics.psb.ugent.be/webtools/Venn/) to identify the intersecting DEGs among the three datasets. Finally, the intersecting DEGs with log_2_FC>2 were deemed to be upregulated genes, and those with log_2_FC<-2 were considered downregulated genes.

### Functional and pathway enrichment analyses

Gene Ontology (GO) analysis 13 and Kyoto Encyclopedia of Genes and Genomes (KEGG) pathway enrichment analysis 14 were performed on the intersecting DEGs using Database for Annotation, Visualization and Integration Discovery (DAVID) version 6.8 15. DEGs enriched in biological processes (BPs), cellular components (CCs) and molecular functions (MFs) were included in the GO analysis (*p<*0.05).

### PPI network construction and module analysis

A PPI network of intersecting DEGs was constructed by an online tool, Search Tool for the Retrieval of Interacting Genes (STRING) 16 version 11.0, to evaluate the protein functions and cellular regulatory mechanisms of DEGs at the molecular level. The Molecular Complex Detection (MCODE) app was a plugin in Cytoscape 17 version 3.7.2 and was applied to check modules of the PPI network. The module with the highest MCODE score is our interesting target.

### Validation of RNA sequencing expression of central genes

Gene Expression Profiling Interactive Analysis (GEPIA; http://gepia.cancer-pku.cn/index.html) and GEPIA2 (http://gepia2.cancer-pku.cn/#index) were the functional tools for analyzing the prevalence and expression of a gene signature in the Cancer Genome Atlas (TCGA) and Genotype-Tissue Expression (GTEx) samples 18. To further validate the intersecting DEGs, GEPIA and GEPIA2 were used to probe the RNA sequencing expression between BC and normal breast tissues. *P<*0.05 was considered significant.

### Survival analysis of central genes

The Kaplan-Meier plotter (KM plotter) system contained gene ChIP and RNA-seq data sources from the TCGA, European Genome-phenome Archive (EGA) and GEO and was generally used for survival analysis and survival biomarker validation 19. In our study, KM plotter was applied to assess the effect of hub genes and miRNAs on survival in BC. The log-rank *p* value and hazard ratio (HR) with 95% confidence intervals were automatically determined and displayed on the plot. A log-rank *p* value <0.05 was considered significant.

### Oncomine™ analysis

Oncomine™ is a web-based data-mining platform consisting of multiple integrated cancer microarray databases and additional clinicopathological data 20. The hub gene-related clinical pathological data of tumors were searched for in this platform to explore the relationship between hub genes and aggressive biological behavior of tumors and analyzed in GraphPad Prism 8. Transcriptional expression of hub genes in 20 different types of cancer diseases was explored in Oncomine™ databases. Differences in transcriptional expression were compared by Student's t-test. The cutoff *p* value was 0.01; the cutoff fold change was 1.5; the gene rank was 10%, and data type was mRNA. Tumor pathological grade and stage of BC were investigated via the Curtis breast dataset (2136 cases), and metastatic event status of BC was studied via the Desmedt breast dataset (198 cases). The threshold of the *p* value was set as 1E-4, and the fold change was 2 in the dataset filter.

### cBioPortal analysis

cBioPortal (www.cbioportal.org) is an open source platform for exploring, visualizing, and analyzing multidimensional cancer genomics and clinical data 21. The dataset we explored is BC (METABRIC, Nature 2012 & Nat Commun 2016) with a total of 2509 samples, among which 1904 samples with complete mutation and copy number alterations were built to analyze the genomic profiles of hub genes. Genetic mutations in hub genes and their association with overall survival (OS) of BC patients were displayed as Kaplan-Meier plots, and the log-rank test was performed to identify the significance of the differences between the survival curves. When a* p* value <0.05, the difference was considered statistically significant.

### Cell culture

A mammary gland epithelial cell line MCF-10A and BC cell lines MCF-7 and MDA-MB-231 were purchased from the Cell Resource Center of the Shanghai Institutes for Biological Sciences. MCF-10A cells were grown in complete growth medium consisting of a 1:1 mixture of Dulbecco's modified Eagle's medium (DMEM) and Ham's F12 medium supplemented with 5% (v/v) horse serum, 10 µg/ml insulin, 100 ng/ml cholera toxin, 20 ng/ml recombinant human epidermal growth factor, 0.5 µg/ml hydrocortisone and 1 unit (U)/ml penicillin/streptomycin. MCF-7 cells and MDA-MB-231 cells were cultured in DMEM (Gibco, Carlsbad, CA, United States) containing 10% fetal bovine serum (Gibco), 1% penicillin (100 U/ml) and 1% streptomycin (100 U/ml). The cells were all incubated in a humidified incubator at 37°C with 5% CO_2_.

### qRT-PCR

Total RNA of the target gene was extracted from the cultured cells using TRIzol reagent (Invitrogen; Thermo Fisher Scientific, Inc.) and quantified using an Ultra-micro UV analyzer Q6000Uv (Quawell Technology, Inc.). The first cDNA strand specific to each gene was synthesized from total RNA using the Bestar™ qPCR RT Kit (cat. no. 2220; DBI Bioscience) on a PCR Amplifier (product no. K960; Hangzhou Jingge Scientific Instrument Co., Ltd.). Real‑time PCR was conducted using Stratagene Mx3000P (Agilent Technologies, Inc.) and by applying Bestar™ qPCR Master Mix (cat. no. 2043; DBI Bioscience). GAPDH was used as an endogenous control and analyzed with the 2-ΔΔCq method. The assay was conducted in triplicate. The primer sequences for each gene are listed in Supplementary Table S1.

### IHC analysis

The Human Protein Atlas (http://www.proteinatlas.org) is a large-scale protein research project, and its main purpose is to map the location of proteins encoded by genes and their expression in human tissues and cells. Each immunohistochemistry (IHC) image in this database has been evaluated by specially educated personnel. We explored the IHC and RNA expression of genes with prognostic value in normal mammary tissues and BC tissues from the Human Protein Atlas.

### Construction of a regulatory network

We generated an miRNA-mRNA regulatory network by using the intersecting DEGs from TarBase version 8 (http://carolina.imis.athena-innovation.gr/diana_tools/web/index.php?r=tarbasev8%2Findex) and miRTarBase (http://mirtarbase.cuhk.edu.cn/php/index.php). The curated TF-miRNA regulations were derived from the TransmiR version 2.0 database (http://www.cuilab.cn/transmir) 22. The following parameters were selected to reduce false positives during processing: (i) number of supporting experiments ≥1 means that at least one high-throughput sequencing of RNA isolated by crosslinking immunoprecipitation (CLIP-Seq) experiment supported the predicted miRNA target site; (ii) up- or downregulated expression patterns between miRNA and mRNA were included; and (iii) level 1 or 2 evidence for supporting the predicted TF in breast tissue was included, and the prediction of literature evidence was excluded. The above interaction information was imported into Cytoscape software version 3.7.2 to construct the TF-miRNA-mRNA regulatory network.

### Statistical analysis

GraphPad Prism 8 (GraphPad Software, Inc.) was used for statistical analyses. The results are expressed as the mean ± standard deviation (SD). One-way analysis of variance and Tukey's post hoc test were used to analyze the differences among three or more groups. A *p* value < 0.05 was considered to indicate a statistically significant difference.

## Results

### Identification of DEGs in BC

**Figure 1:**
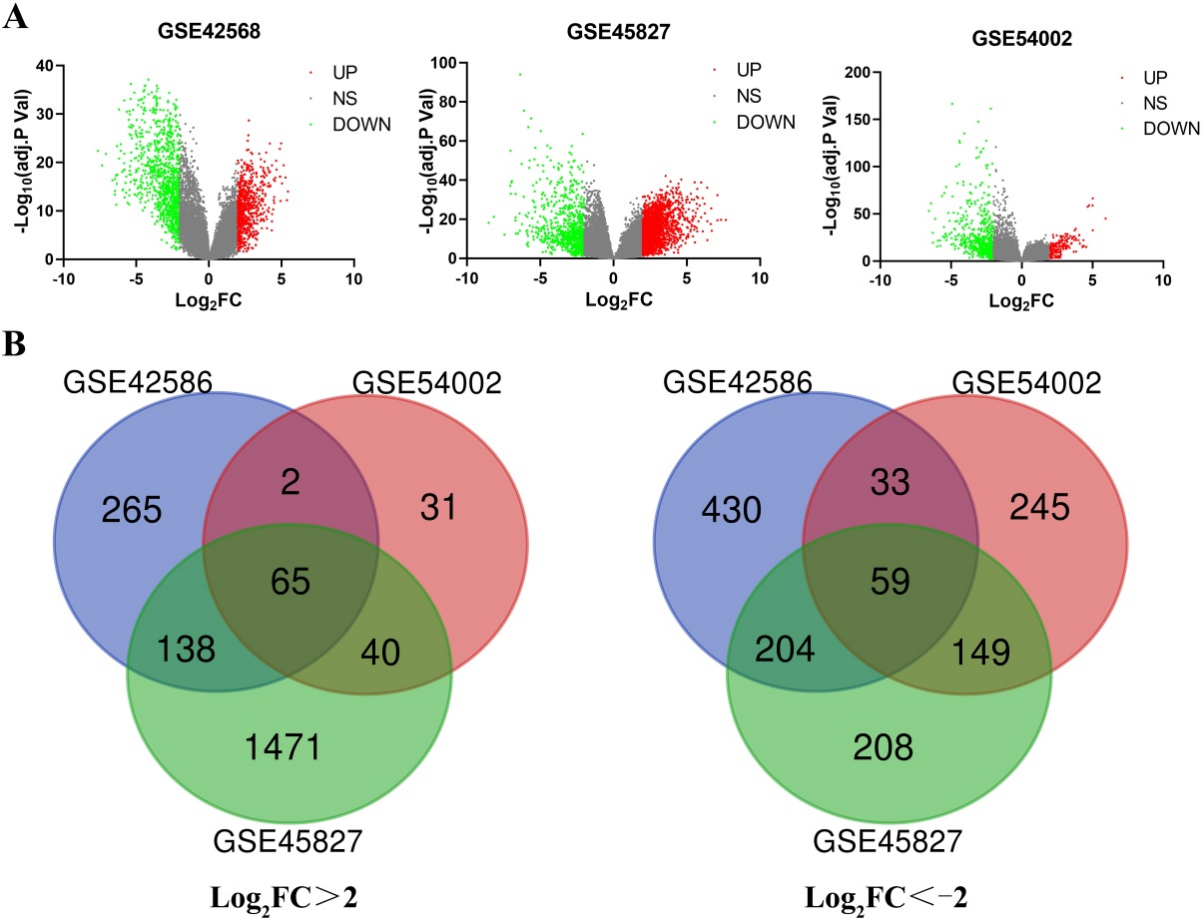
Identification of the DEGs in three datasets (GSE42568, GSE45827 and GSE54002). (A) The volcano map for each dataset; red dots indicate upregulated DEGs; green dots represent downregulated DEGs; and gray dots indicate nonsignificant (NS) DEGs. (B) A total of 124 common DEGs were identified in an online Venn diagram tool. Different colors represent different datasets. Sixty-five overlapping upregulated DEGs in the three datasets (log_2_FC > 2) and 59 overlapping downregulated DEGs in the three datasets (log_2_FC < -2) were identified.

A total of 651 BC tissues and 44 normal breast tissues were included in our study. The series from each chip, or sample, was separately analyzed via R software, resulting in the list of DEGs. GSE42568, GSE45827 and GSE54002 chips were screened, resulting in 1196 (470 upregulated and 726 downregulated), 2334 (1714 upregulated and 620 downregulated) and 624 (138 upregulated and 486 downregulated) DEGs, respectively (Figure 1A). After using an online Venn diagram software, a total of 65 upregulated DEGs (logFC>2) and 59 downregulated DEGs (logFC<-2) that overlapped in the BC tissues were extracted and identified from these three chips (Figure 1B and Supplementary Table S2).

### GO function and KEGG pathway enrichment analyses of DEGs

All 124 overlapping DEGs were further used to explore the biological functions via DAVID software. The BP results suggested that upregulated DEGs were significantly enriched in collagen fibril organization, regulation of cell cycle, mitotic sister chromatid segregation and mitotic cytokinesis (Supplementary Figure S2A), and downregulated DEGs were mainly enriched in negative regulation of anoikis, insulin receptor signaling pathway, cellular response to starvation and positive regulation of cell proliferation (Supplementary Figure S2B). MF analysis indicated that upregulated DEGs were significantly enriched in proteinaceous extracellular matrix, midbody, spindle microtubule, cytoplasm and nucleus (Supplementary Figure S2C), and downregulated DEGs were mainly enriched in extracellular space, membrane raft, extracellular region, focal adhesion and proteinaceous extracellular matrix (Supplementary Figure S2D). CC results revealed that upregulated DEGs were significantly enriched in ATP binding, heparin binding, extracellular matrix structural constituent and microtubule motor activity (Supplementary Figure S2E), and downregulated DEGs were mainly enriched in heparin binding, signal transducer activity, protein binding, bridging and metalloendopeptidase activity (Supplementary Figure S2F).

KEGG pathway analysis showed that upregulated DEGs were significantly enriched in cell cycle, extracellular matrix (ECM)-receptor interaction, oocyte meiosis, p53 signaling pathway and protein digestion and absorption (*p<*0.05), while downregulated DEGs displayed no significantly enriched signaling pathways (Supplementary Table S3).

### PPI network construction and modular analysis

**Figure 2:**
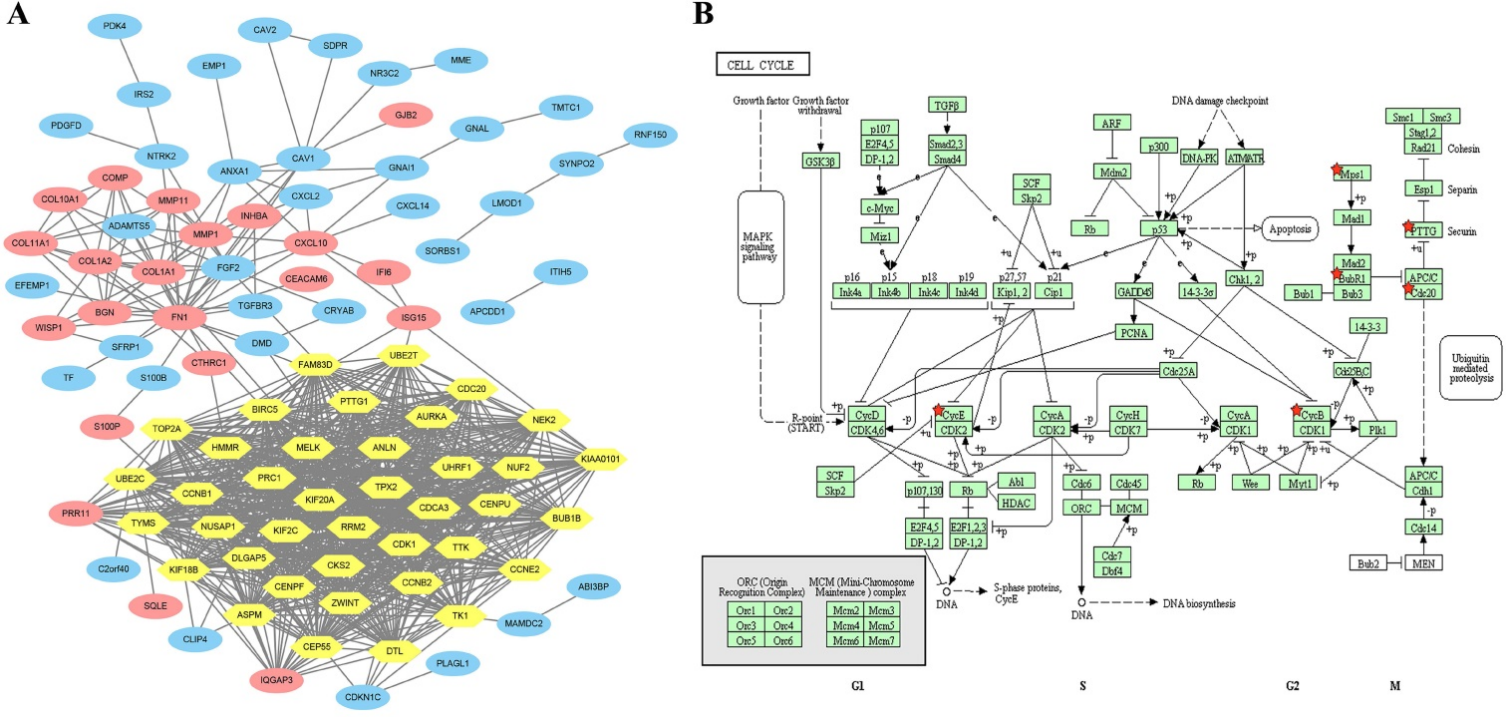
Overlapping DEGs constructed from the PPI network by STRING and MCODE plugin app were analyzed again with the KEGG enrichment pathway. (A) A total of 97 DEGs are involved in the PPI network complex. The nodes represent proteins, and the edges indicate the interaction of proteins. Blue circles represent downregulated DEGs, and red and yellow circles represent upregulated DEGs. Yellow circles are identified with the highest score by MCODE module analysis (degree cutoff= 2, node score cutoff= 0.2, k-core = 2, and max. depth = 100). (B) Seven genes (*CCNE2*, *CCNB1*, *CCNB2*, *BUB1B*, *TTK*, *CDC20* and *PTTG1*, labeled with a red star) were significantly enriched in the cell cycle pathway, especially in G2/M phases. *Mps1* represents *TTK*; *BubR1* represents *BUB1B*; *CycB* represents both *CCNB1* and *CCNB2*; and *CycE* represents *CCNE2*.

A total of 124 overlapping DEGs were mapped with STRING to explore potential interactions of the DEGs at the protein level. Ninety-seven nodes and 881 edges were constructed and represented in the PPI network with a PPI enrichment *p* value <1.0E-16 and included 60 upregulated and 37 downregulated DEGs (Figure 2A). A significant module was subsequently applied and constructed with 39 nodes and 709 edges, which gained the highest score in MCODE (yellow part in Figure 2A). Interestingly, the 39 central nodes were all upregulated genes in BC tissues.

### Expression analysis of core genes via GEPIA and survival analysis via KM plotter

The 39 core genes were imported into the online GEPIA analysis tool with |log_2_FC| cutoff value=1 and *p* value cutoff value=0.01. The results showed that these 39 core genes were all significantly expressed in BC patients compared with patients with normal breast tissue (Supplementary Figure S3 and Supplementary Table S4). To further clarify the significance of these 39 core genes for BC survival, KM plotter was applied for analysis. It turned out that 36 out of the 39 upregulated genes showed a markedly worse survival (*p<*0.05, Supplementary Figure S3 and Supplementary Table S5).

### KEGG pathway enrichment of the 36 selected core genes

A total of 36 selected core genes were analyzed again using KEGG pathway enrichment analysis by DAVID to explore the vital functions driving tumorigenesis in BC. The results indicated that seven hub genes (cyclin E2 (*CCNE2*), cyclin B1 (*CCNB1*), cyclin B2 (*CCNB2*), mitotic checkpoint serine/threonine kinase B (*BUB1B*), dual-specificity protein kinase (*TTK*), cell division cycle 20 (*CDC20*), and pituitary tumor transforming gene 1 (*PTTG1*)) were significantly enriched in the cell cycle pathway (*p*=1.42E-07, Figure 2B and Supplementary Table S6).

### Transcripts and survival analysis of hub genes

**Figure 3:**
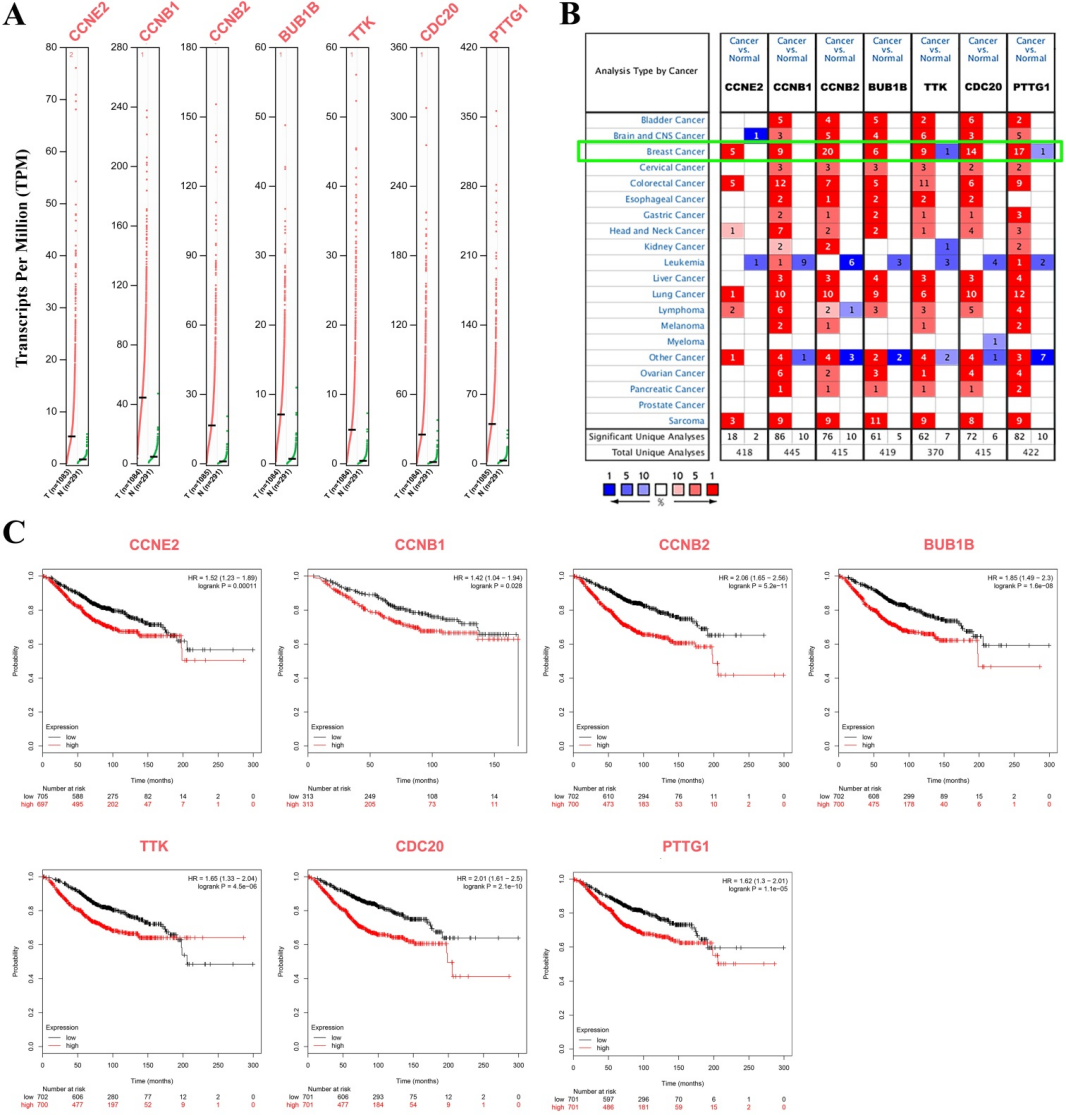
Transcription and survival analysis of hub genes. (A) Transcription levels of seven cell cycle-related hub genes via GEPIA (*p<*0.05); red dots represent BC samples, and green dots represent normal tissue samples. (B) Transcriptional expression of seven CCRGs was significantly high in BC (*p*<0.01, green frame, Oncomine™ database). Numbers represent the significantly differential expression datasets. (C) Survival analysis of seven CCRGs via KM plotter (*p<*0.05); the red line represents high expression, and the dark line represents low expression in BC patients.

Seven cell cycle-related genes (CCRGs) were explored again at the transcript level via GEPIA and confirmed via the Oncomine™ database. The results showed that *CCNE2, CCNB1, CCNB2, BUB1B, TTK, CDC20* and* PTTG1* were significantly highly expressed in BC patients (*p*<0.05, Figure 3A) and confirmed that the transcriptional expression of these seven CCRGs was significantly higher in the BC population (*p*<0.01, green frame, Figure 3B) than in the normal population. KM plotter analysis indicated that high expression of these seven CCRGs led to an unfavorable prognosis (*p<*0.05, Figure 3C).

### Analysis of clinicopathological characteristics of hub genes

**Figure 4:**
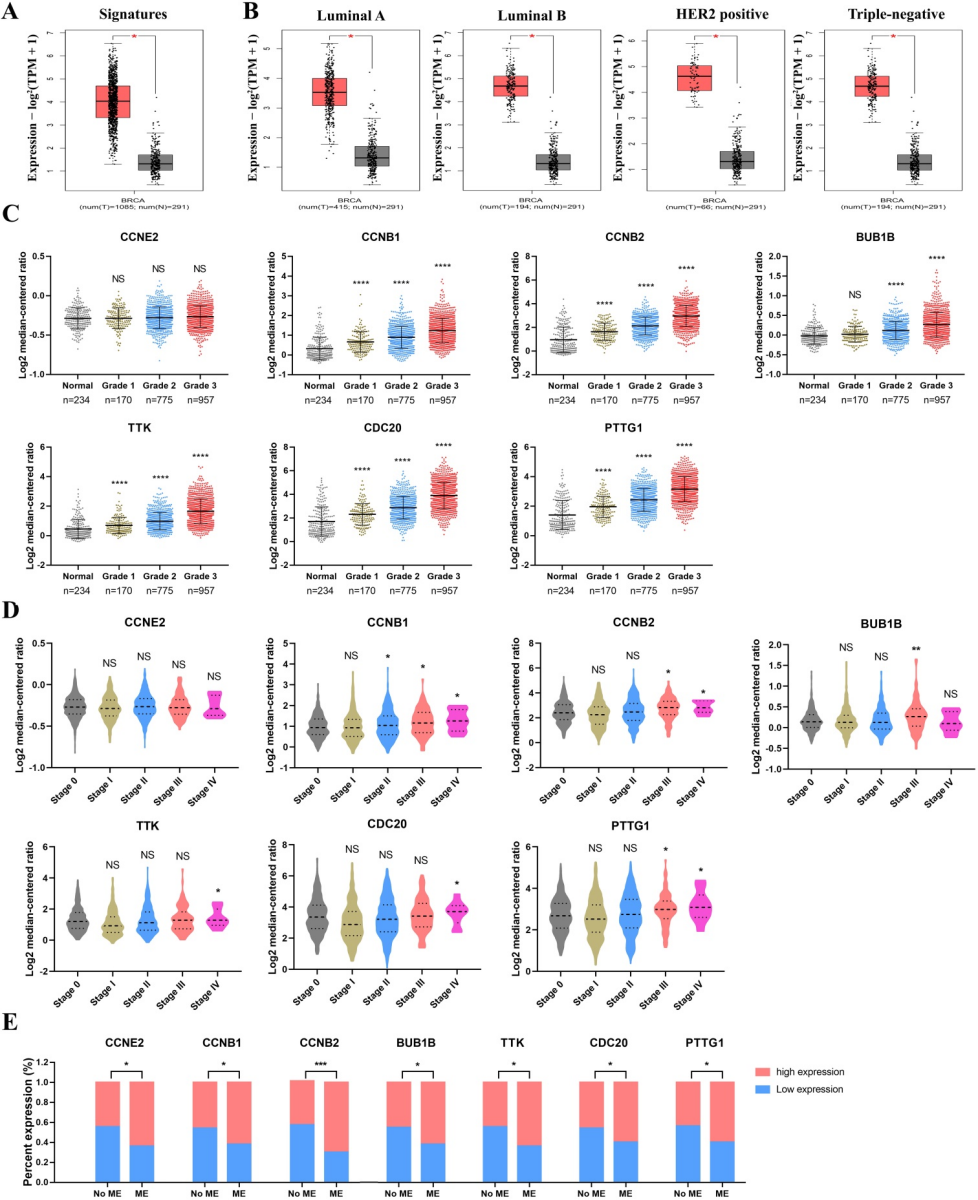
Analysis of the clinicopathological characteristics of hub genes. (A) The expression level of the CCRG set in BC and normal patients; the red box represents tumor tissues, and the gray box represents normal tissues. (B) The expression level of the CCRG set in four BC subtypes (luminal A, luminal B, HER2-positive and TNBC); the red box represents tumor tissues, and the gray box represents normal tissues. (C) The relation between CCRGs and pathological grade. (D) The relation of CCRGs and tumor stages; n=492, 372, 579, 90 and 10 in stages 0, I, II, III and IV; (E) the relation between the expression level of seven CCRGs and metastatic events (MEs). NS means nonsignificant, ^*^p<0.05, ^**^p<0.01, ^***^p<0.001 and ^****^p<0.0001.

Seven CCRG signatures were processed as a CCRG set, and correlation analysis of BC subtypes and stages between BC and normal patients was performed via GEPIA2. As shown in Figure 4A, the expression level of the CCRG set was obviously high in BC patients, and the difference was statistically significant (*p<*0.05). Similarly, analysis of the BC subgroups demonstrated that the CCRG set was significantly highly expressed in the four BC subtypes (luminal A, luminal B, HER2-positive and triple-negative (TNBC)) (*p<*0.05, Figure 4B). High expression of CCRGs was significantly associated with tumor pathological grade (*p<*0.05), indicating that a higher grade accompanied higher expression of CCRGs, except for the *CCNE2* gene (Figure 4C). In the clinical tumor stage study, the worse prognosis of stage III and IV tumors was associated with higher expression of CCRGs (*p<*0.05, respectively), except for the *CCNE2* gene (Figure 4D). Metastatic event analysis showed that an elevated expression level of CCRGs was significantly related to the metastasis event (*p<*0.05, Figure 4E).

### Genetic mutations in CCRGs and their association with copy number alteration frequency and OS of BC patients

**Figure 5:**
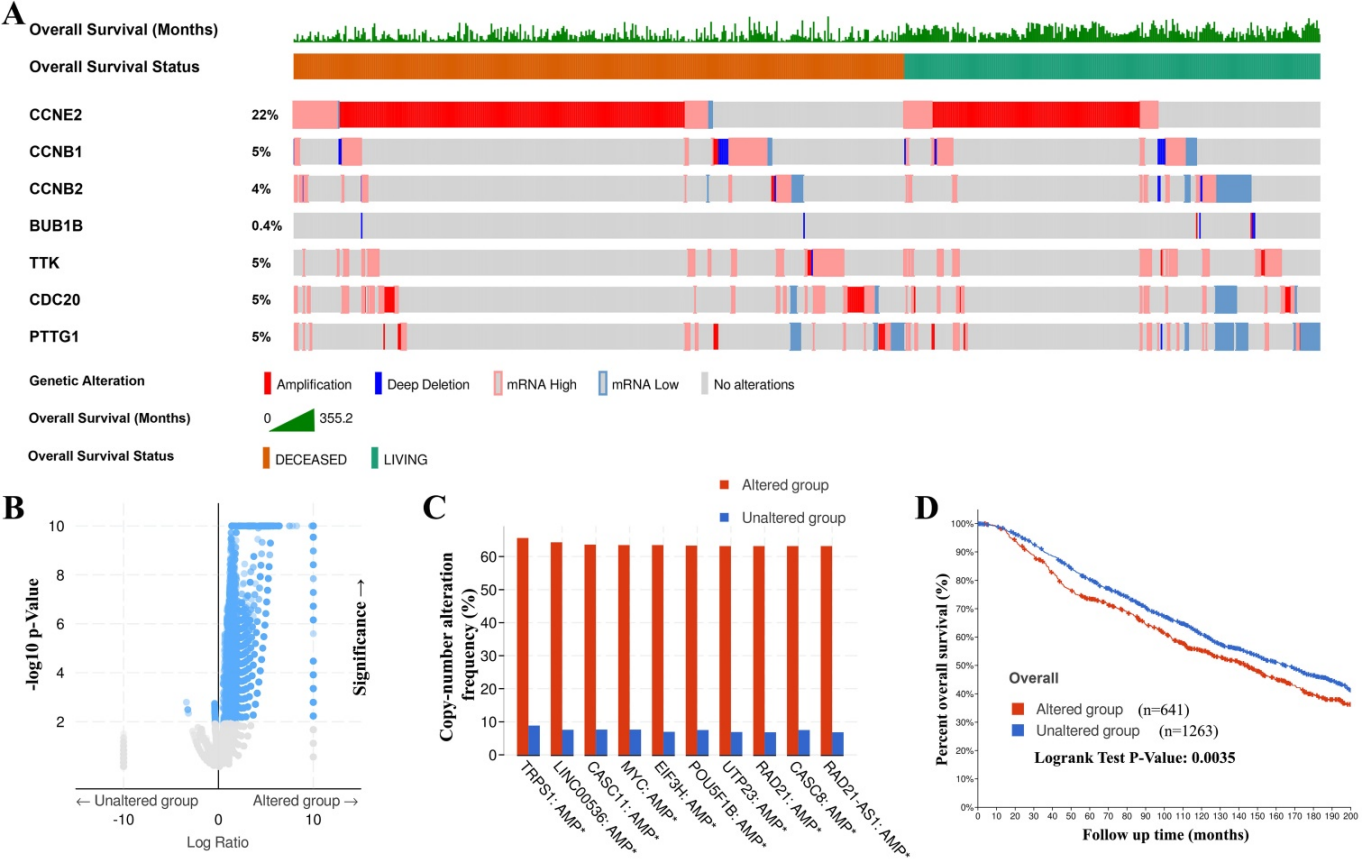
Genetic mutations in CCRGs and their associations with copy number alteration frequency and OS of BC patients (cBioPortal). (A) A high mutation rate (43%, 641/1904) of CCRGs was observed in BC patients. CCNE2 was ranked as the gene with the highest number of genetic alterations, with a 22% mutation rate. (B) Putative DNA copy number alterations showed more genes with copy number alterations clustered in the altered group, and (C) the top 10 genes with the highest alteration frequencies were markedly enriched in the altered group. (D) Genetic alterations in CCRGs were associated with shorter OS in BC patients.

The genetic alteration in CCRGs and their association with copy number alteration frequency and OS of BC patients were probed via cBioPortal. As shown in Figure 5A, a high mutation rate of CCRGs was observed in BC patients. In 1904 gene sequences with complete information for BC patients, genetic alterations were found in 641 BC patients, and the mutation rate was 34%. *CCNE2* was ranked as the gene with the highest number of genetic alterations, and the mutation rate was 22%. Then, genetic alterations of CCRGs in 641 patients were classified as the altered group, and the remaining 1263 patients were classified as the unaltered group. Further study revealed that more genetic mutations synergistically occurred in the altered group (Figure 5B), and the top 10 genes with the highest alteration frequency were markedly enriched in the altered group (Figure 5C). Kaplan-Meier plots and log-rank tests showed that genetic alterations in CCRGs were associated with shorter OS (Figure 5D, *p*=0.0035) in BC patients. These results implied that genetic alteration of CCRGs could also significantly affect BC patients' prognosis.

### Expression levels of CCRGs in BC cell lines

**Figure 6:**
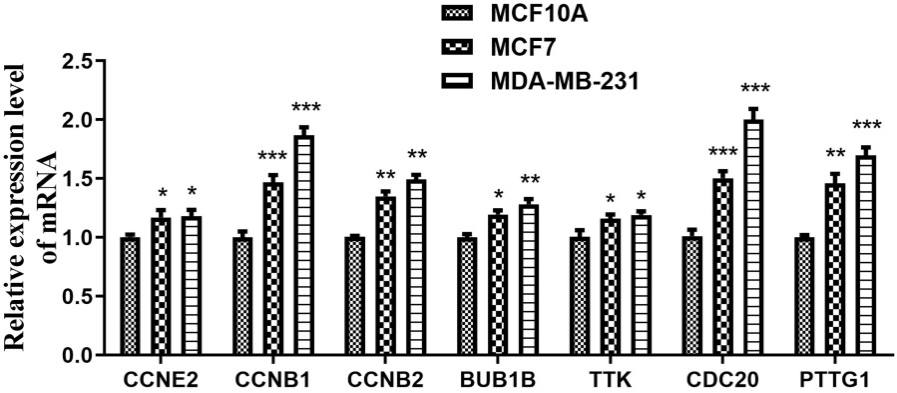
Expression of seven hub genes in the mammary gland epithelial cell line and BC cell lines. The expression levels of *CCNE2*, *CCNB1*, *CCNB2*, *BUB1B*, *TTK*, *CDC20* and *PTTG1* were significantly higher in the BC cell lines (MCF-7 and MDA-MB-231) than in the MCF-10A cell line. ^*^p<0.05, ^**^p<0.01 and ^***^p<0.001.

Next, qRT-PCR assays were performed to detect the expression levels of the seven CCRGs in the MCF-10A, MCF-7 and MDA-MB-231 cell lines. The results shown in Figure 6 indicated that *CCNE2, CCNB1, CCNB2, BUB1B, TTK, CDC20* and* PTTG1* were all highly expressed in BC cell lines (MCF-7 and MDA-MB-231), and there were significant differences (*p<*0.05) when compared to the MCF-10A cell line.

### Validation of CCRGs in clinical tissue samples

**Figure 7:**
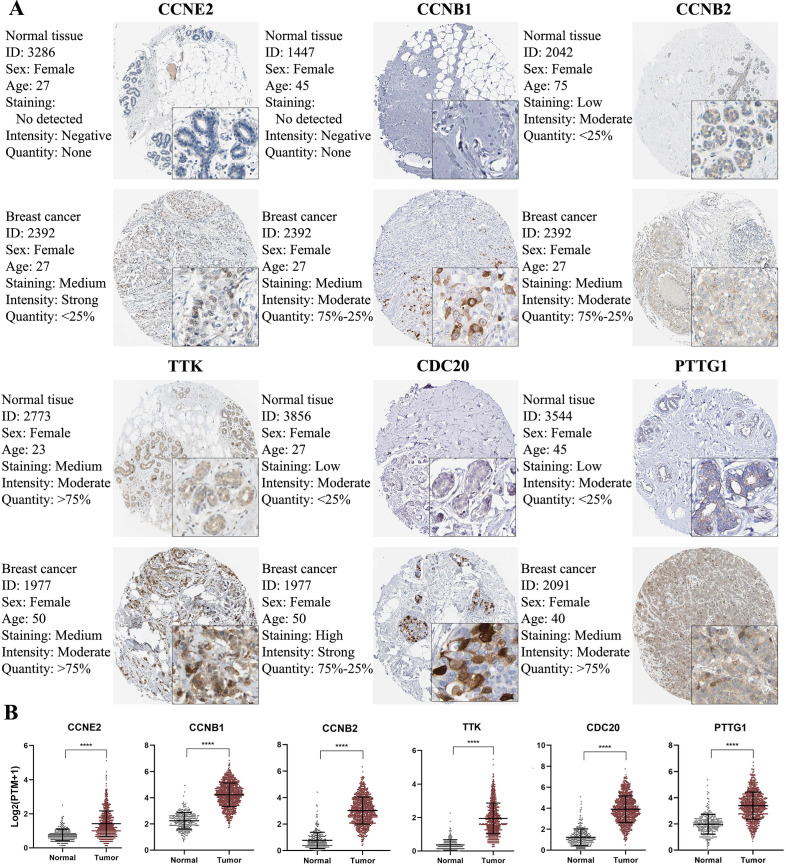
IHC analysis and RNA expression analysis of genes with prognostic values. (A) Differentially expressed proteins of genes with prognostic values in normal and BC tissues in the Human Protein Atlas database. (B) RNA expression of genes with prognostic values between normal and BC tissues in the Human Protein Atlas database. Significance was tested by the Student's t‑test (^****^p < 0.0001; number of normal samples and tumor samples were 290 and 1075, respectively).

To further confirm the protein expression of CCRGs in BC, we used IHC to compare the protein expression between normal mammary tissues and BC tissues via the Human Protein Atlas database. The IHC map of CCRGs is shown in Figure 7 and confirmed that *CCNE2, CCNB1, CCNB2, TTK, CDC20* and* PTTG1* were significantly overexpressed in BC tissues compared with normal mammary tissues (*p<*0.05); however, *BUB1B* was not confirmed to be significantly overexpressed in BC tissues due to the lack of data available in the Human Protein Atlas.

### TF-miRNA-mRNA regulatory network

**Figure 8:**
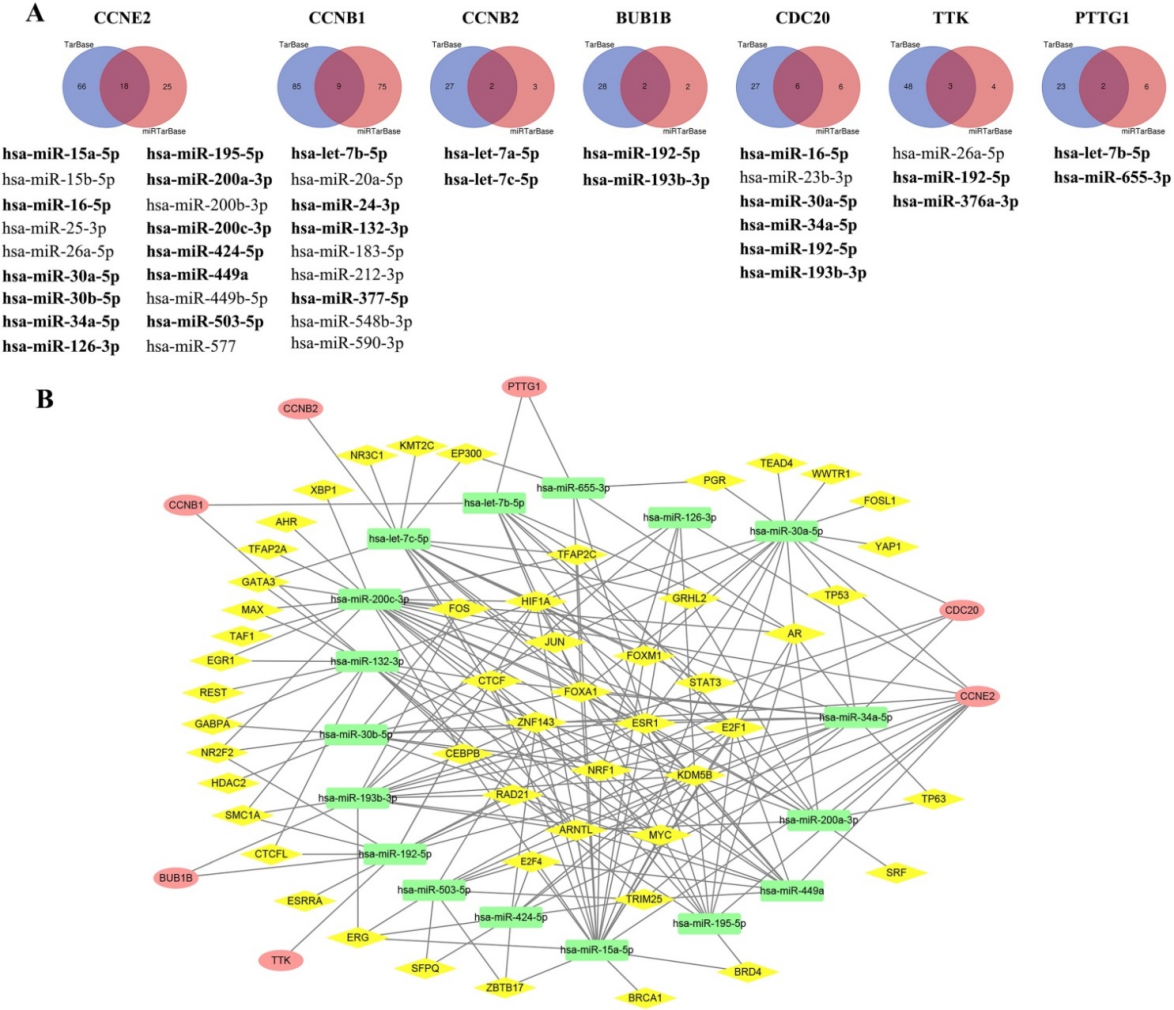
Identification of predicted miRNAs targeted to CCRGs and construction of the TF-miRNA-mRNA regulatory network. (A) Using the intersecting DEGs from TarBase and miRTarBase to select predicted miRNAs and confirm significant expression of miRNAs via the KM plotter (bold miRNAs). (B) Construction of the TF-miRNA-mRNA regulation network via the integration of published multilevel expression data and a bioinformatics computational approach; red oval indicates mRNA of CCRGs, yellow diamond represents TF and green rectangle means miRNA.

Using the KM plotter, we obtained a total of 34 predicted miRNAs targeted to CCRGs (Figure 8A) and confirmed that the expression of 22 out of 34 miRNAs significantly affected BC patient survival (bold miRNAs in Figure 8A and Supplementary Figure S5). Of these, 17 miRNAs with TF binding sites were used to construct the miRNA-mRNA regulatory network (Figure 8B, Supplementary Table S7). The top ten regulating TFs were KDM5B, ARNTL, E2F1, HIF1A, ESR1, FOXA1, CTCF, NRF1, MYC and AR.

## Discussion

Numerous studies have revealed that specific genes involved in the cell cycle may play a critical role in the biology of BC and may be of clinical relevance in BC, knowledge which should assist in improving disease prognoses and therapy 23. In our present work, seven potentially prognostic biomarker genes (*CCNB2*, *CCNB1*, *CDC20*, *PTTG1*, *BUB1B*, *TTK* and *CCNE2*) that were remarkably enriched in the cell cycle pathway and significantly associated with BC prognosis were screened and evaluated via bioinformatics methods. These genes were confirmed and further analyzed in the Oncomine^TM^ platform, indicating that the high expression of seven CCRGs was significantly related to the aggressive biological behavior of tumors, which manifested as a higher level of pathological grade and stage and were more prone to metastasis. Genetic mutation analysis revealed that genetic alteration of CCRGs could also contribute to poor prognosis of BC patients. Verification by qRT-PCR assay in BC cell lines and by IHC in clinical tissue samples further supports the bioinformatics results. Construction of the TF-miRNA-mRNA regulatory network reveals the potential mechanism of CCRGs participating in tumorigenesis and indicates that targeting cell cycle treatment would be a potentially effective choice for BC patients to have a more satisfactory prognosis.

*CCNB2*, a member of the cyclin family of proteins, which cyclin B1 and cyclin B2, binds to cyclin-dependent kinases (CDKs) and regulates the activity of *CDKs* and different cyclin functions in specific phases of the cell cycle 24. Abnormal expression of *CCNB2* leads to G2/M checkpoint failure during the cell cycle, which may create gene mutations and carcinogenesis 25. *CCNB1*, a highly conserved member of the cyclin family of proteins, is expressed in almost all tissues of the human body 26 and is a key monitoring protein in controlling cell cycle progression from the G2 phase to mitosis by regulating cyclin dependent kinase 1 (*CDK1*) 27, 28. Studies have indicated that *CCNB1* is also involved in some biological behaviors, such as apoptosis, chemoresistance, and epithelial-mesenchymal transition of tumor cells 29, 30. *CDC20* is an essential cell cycle regulator that drives mitosis from metaphase to anaphase by activating the anaphase-promoting complex 31. A study revealed that *CDC20* mutations blocked cell division and stopped cell cycle progression toward anaphase and chromosome segregation 32. Some reports have indicated that *CDC20* plays an oncogenic role in human tumorigenesis and that genetic ablation of *CDC20* blocks *in vivo* tumorigenesis 33, 34. *PTTG1*, also known as securin, is located on chromosome 5 (5q35.1) and is involved in regulating sister chromatid separation and the transition from metaphase to anaphase 35, 36. In-depth research indicated that *PTTG1* acts as an oncogene and plays an important role in promoting cell cycle progression, sustaining chromosomal stability, and modulating transformation *in vitro* and tumorigenesis *in vivo* 37-39. *BUB1B* is essential for controlling mitotic timing, and its main functions in mitosis include activation, maintenance, and silencing of the spindle assembly checkpoint protein as well as regulating chromosome-spindle attachment 40, 41. *BUB1B* was reported to interact with *CDC20* directly and activate the anaphase-promoting complex/cyclosome by inhibiting *CDC20* activity to ensure proper chromosome segregation by blocking the initiation of anaphase 42.* TTK*, also known as monopolar spindle 1 (*Mps1*), is crucial for the mitotic checkpoint and ensures exact chromosome segregation and proper attachments 43-45. A higher abundance of mitotic checkpoint genes in cancer cells is markedly associated with increased genome instability and is even correlated with tumor cell spread and cancer metastasis 46, 47. *CCNE2* is a member of the cyclin family of proteins and plays a role in the cell cycle by regulating the G1 to S phase transition to ensure cell division 48. Upregulated *CCNE2* in various cancers was shown to be correlated with tumorigenesis and tumor proliferation, invasion, and migration by affecting tumor cell viability and apoptosis 49.

The cell cycle, also known as the cell-division cycle, is a series of processes that occur in cell progression, resulting in genome duplication and cell division to produce two daughter cells 23; several checkpoints are required to maintain genomic stability and decrease the possibility of tumorigenesis. Cyclins and *CDKs* are involved in and run through the whole cell cycle, and their expression levels and effects occur across specific phases 23, 50. Ignoring the many safeguards and checkpoints of the cell cycle, cancer cells start to exhibit limited proliferation regardless of their aneuploidy and other cellular defects. This phenotype is achieved through alterations of various genetic and epigenetic molecules that hyperactivate or inactivate key components of the cell cycle 51. Different subtypes of BC display different molecular alterations and dependencies on the cell cycle and its checkpoints 52; for example, multiple studies have revealed that TNBC tumors are dependent on the spindle assembly checkpoint and showed high expression levels of mitotic checkpoint genes (e.g., *TTK* and *BUB1B*) 53, 54. Given that dysregulation of the cell cycle by aberrant activation of cyclins or other mitotic checkpoint genes is essential for cancer cell proliferation, targeting the cell cycle is a promising anticancer therapeutic strategy 55.

Accumulating evidence has demonstrated that genetic mutations are associated with the occurrence and prognosis of tumors 56-58. In the present study, it was identified that mutations in CCRGs were associated with the biological features of BC. Mutations in CCRGs were identified to affect OS. Notably, these genes may represent potential novel biomarkers for assessing the prognosis of patients with BC and may be considered potential targets for treating BC.

MiRNAs, a type of noncoding RNA with 21-25 nucleotides, play a critical role in regulating the expression of mRNAs by forming the RNA-induced silencing complex (RISC) to inhibit the translation or degrade the mRNA directly. Seventeen miRNAs targeting CCRGs were identified and may be a part of the mechanism by which CCRGs participate in tumor aggressive BPs. Studies have revealed that miRNA expression is related to transcriptional TFs 59,60. However, the involvement of several miRNAs and TFs in the transcriptional regulation of mRNA and miRNAs has rarely been reported in BC. Therefore, we firstly constructed a TF-miRNA-mRNA regulatory network in BC, which consists of CCRGs. This study provides new insight into the molecular mechanism of BC.

Abundant studies and our results have demonstrated that seven CCRGs contribute to the aggressive biological behavior of tumors. Some of these CCRGs have been suggested to be significantly related to poor prognosis in BC based on clinical research; however, some of these CCRGs, namely, *PTTG1*, *TTK* and *CCNE2*, have not. In addition, some of these CCRGs have been used as antitumor targets or suggested to be related to therapeutic resistance in BC; however, some of them, including *CCNB2*, *CDC20*, *PTTG1* and *BUB1B*, have not yet been determined as antitumor targets or to be related to therapeutic resistance in BC. Furthermore, the expression level of seven CCRG mRNAs was significantly high in BC cell lines via qRT-PCR, suggesting that they might be effective agents for BC treatment. We attempted to reveal the potential mechanism driving the CCRG-induced uncontrolled cell cycle in BC by constructing a TF-mRNA-mRNA regulatory network. However, further in-depth studies are still urgently needed. Our data could theoretically lay the foundation and provide a significant direction for future investigations of these seven CCRGs to investigate the aggressive biological behavior of tumors and target cell cycle treatments in BC.

## Conclusion

In summary, this bioinformatics analysis study determined seven CCRGs (*CCNB2*, *CCNB1*, *CDC20*, *PTTG1*, *BUB1B*, *TTK* and *CCNE2*) with poor prognosis in BC based on tissue-specific microarray datasets. The results indicated that these seven CCRGs all played a part in the aggressive biological behavior of tumors by regulating the cell cycle, and this study is the first to construct a TF-miRNA-mRNA regulatory network for CCRGs in BC. This study provides new insights into the molecular mechanism of BC.

## Supplementary Material

Supplementary figures and tables.
