# Supplementary material for: Identification of Seven Cell Cycle-Related Genes with Unfavorable Prognosis and Construction of their TF-miRNA-mRNA regulatory network in Breast Cancer
Source: J Cancer. 2021 Jan 1;12(3):740–53. doi: 10.7150/jca.48245 (PMC7778540; doi:10.7150/jca.48245)
Supplement: Supplementary file 1 — Supplementary figures and tables. [file jcav12p0740s1.pdf]

Supplementary Figures

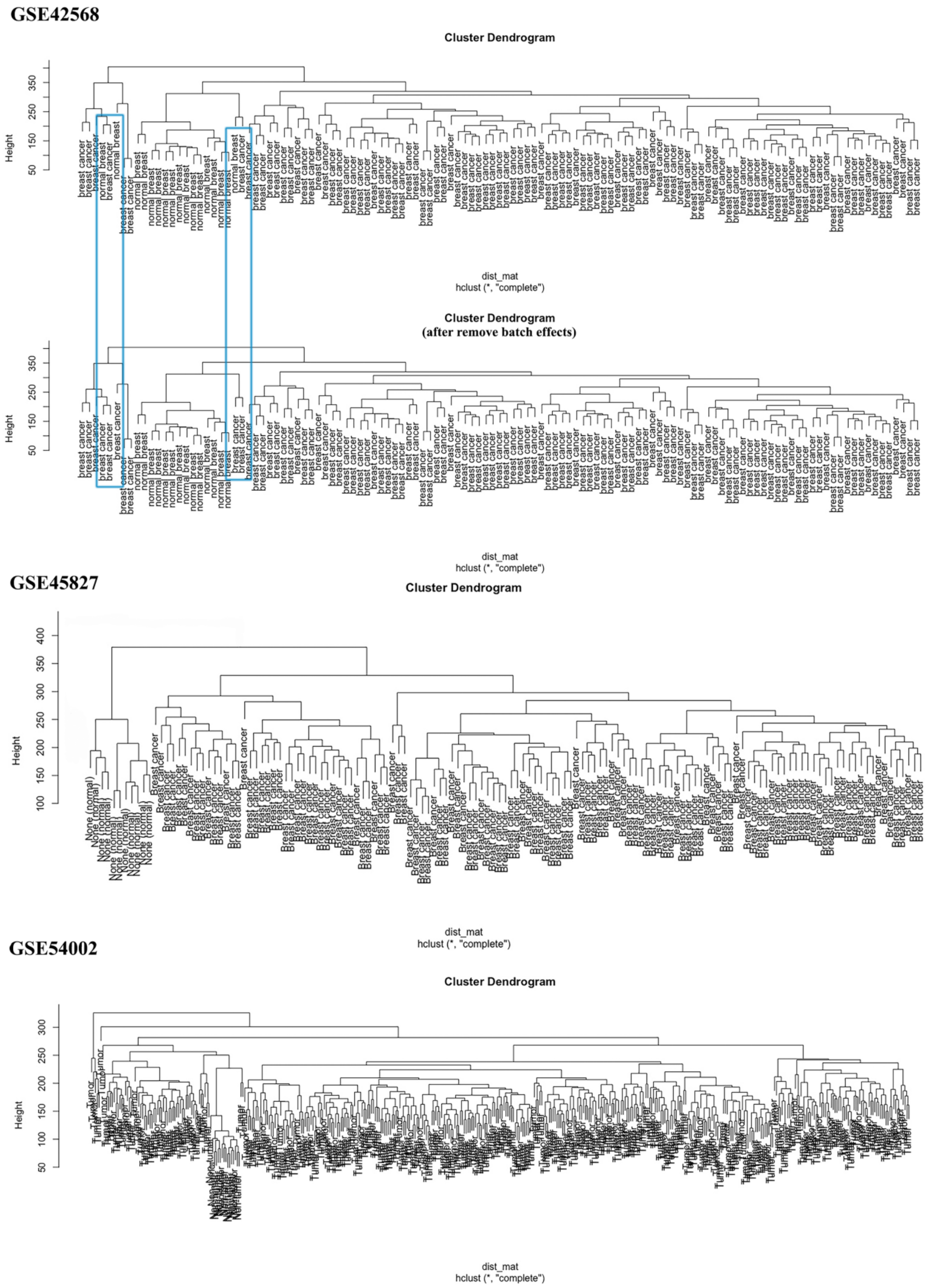

Figure S1 Identification and correction batch effects in each dataset.

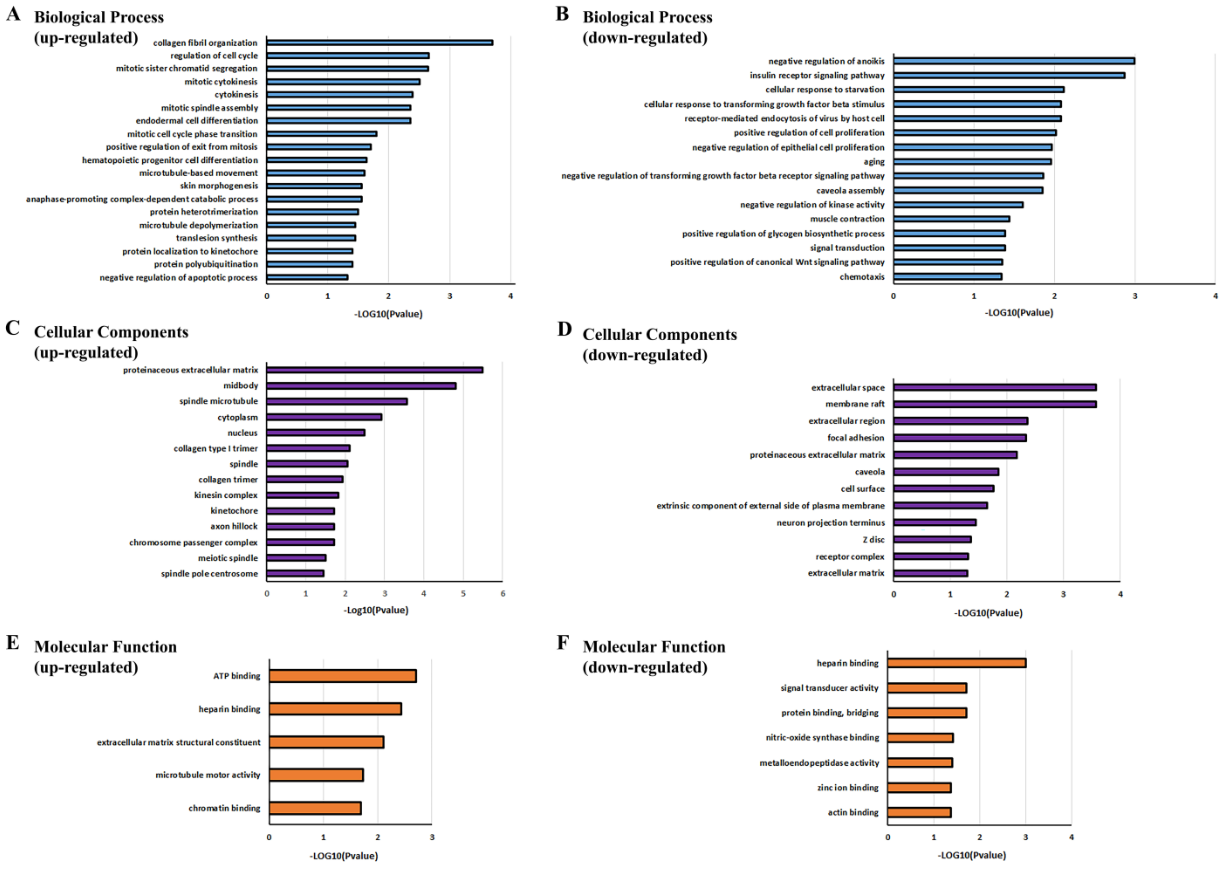

Figure S2 Top 10 functional analysis of the overlapping DEGs. (A) Biological process enrichment of up-regulated DEGs; (B) Biological process enrichment of down-regulated DEGs; (C) Molecular function enrichment of up-regulated DEGs; (D) Molecular function enrichment of down-regulated DEGs; (E) Cellular component enrichment of up-regulated DEGs; (F) Cellular component enrichment of down-regulated DEGs.

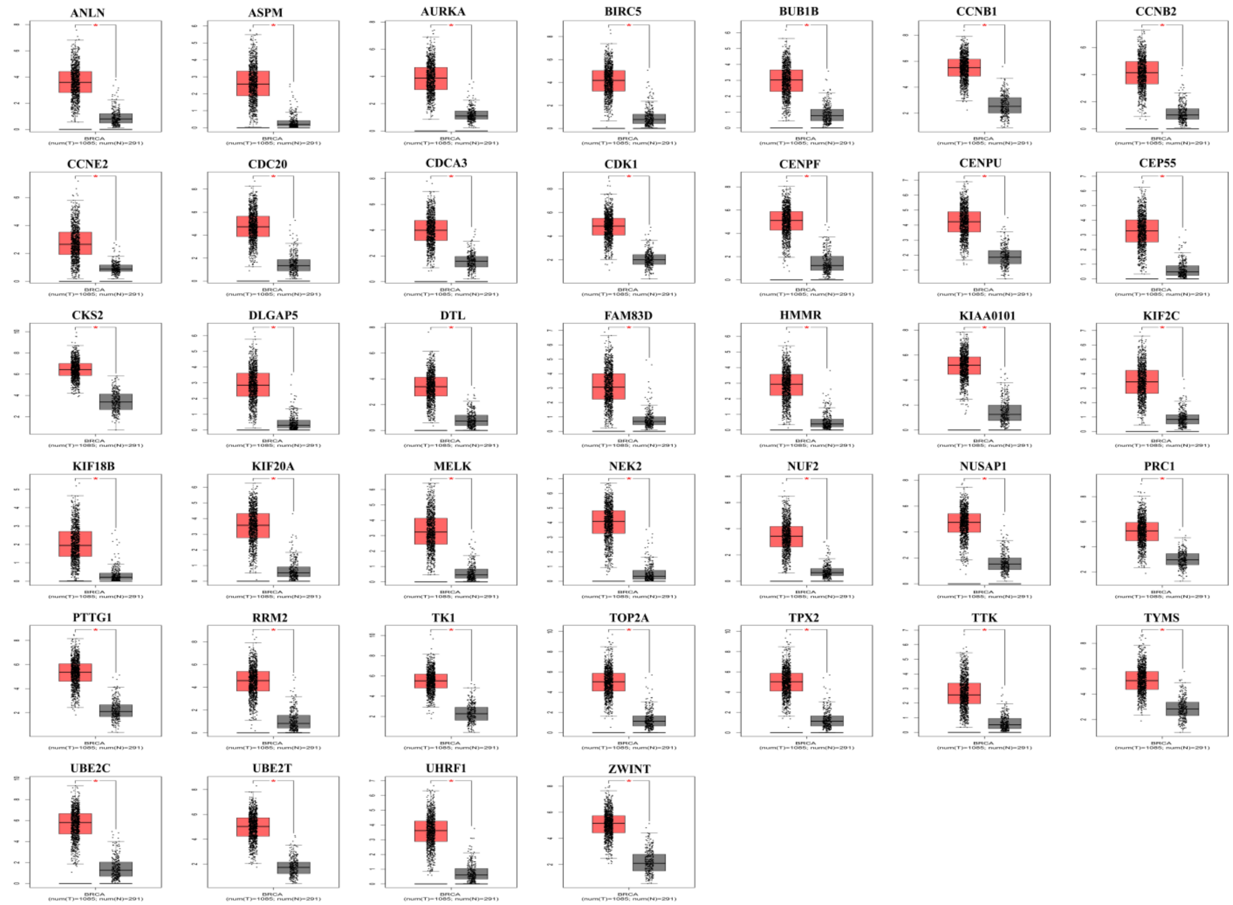

Figure S3 A total of 39 central DEGs showed significantly high expression in BC patients compared to healthy people (*$p < 0.05$). Red box represent tumor tissues and grey box means normal tissues.

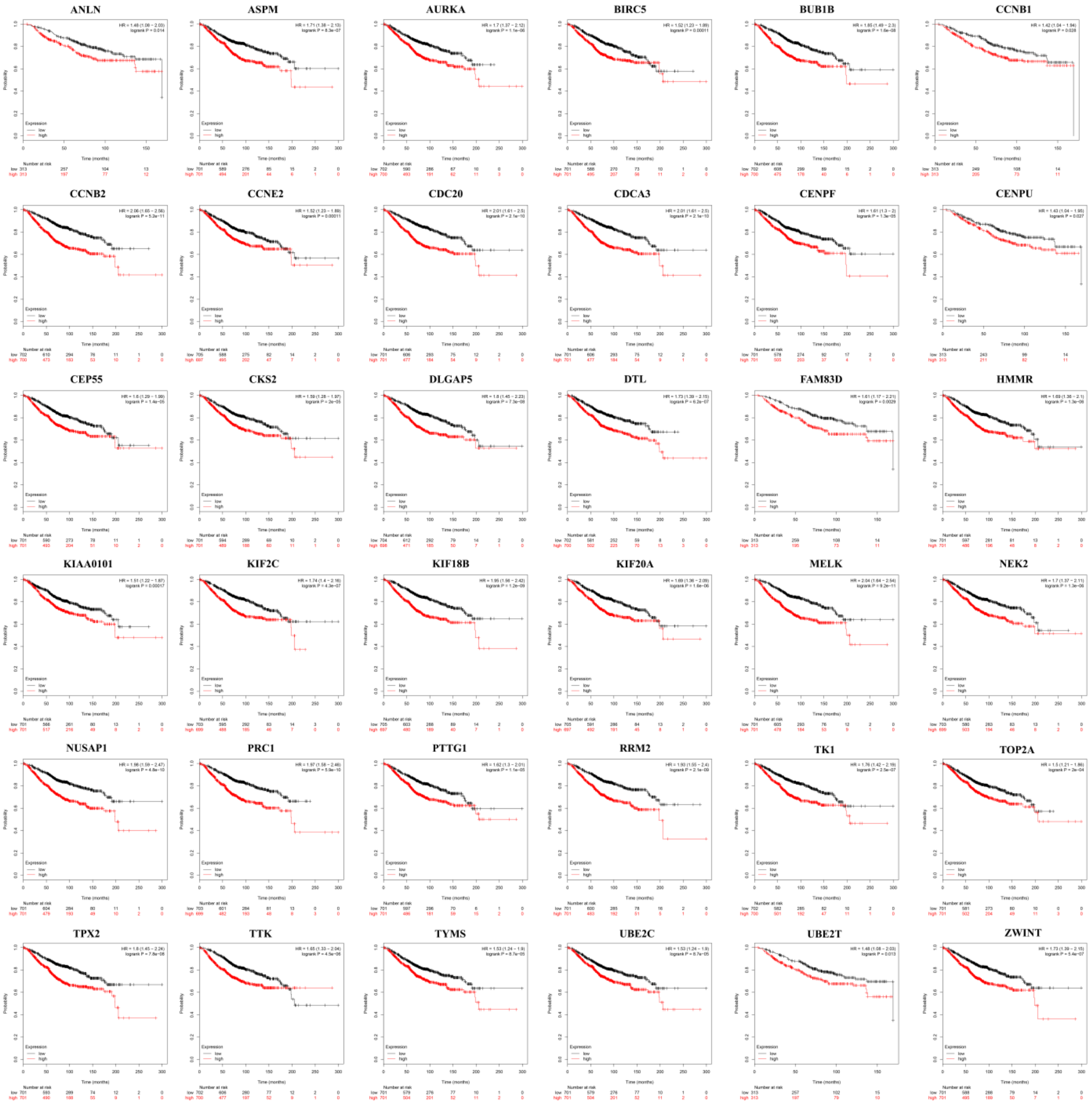

Figure S4 The 39 central DEGs were imported to Kaplan-Meier plotter online tools and 36 out of 39 showed significantly related to worse prognosis in BC patients ($p < 0.05$). Red lines represent high expression genes and black lines means low expression genes in BC patients.

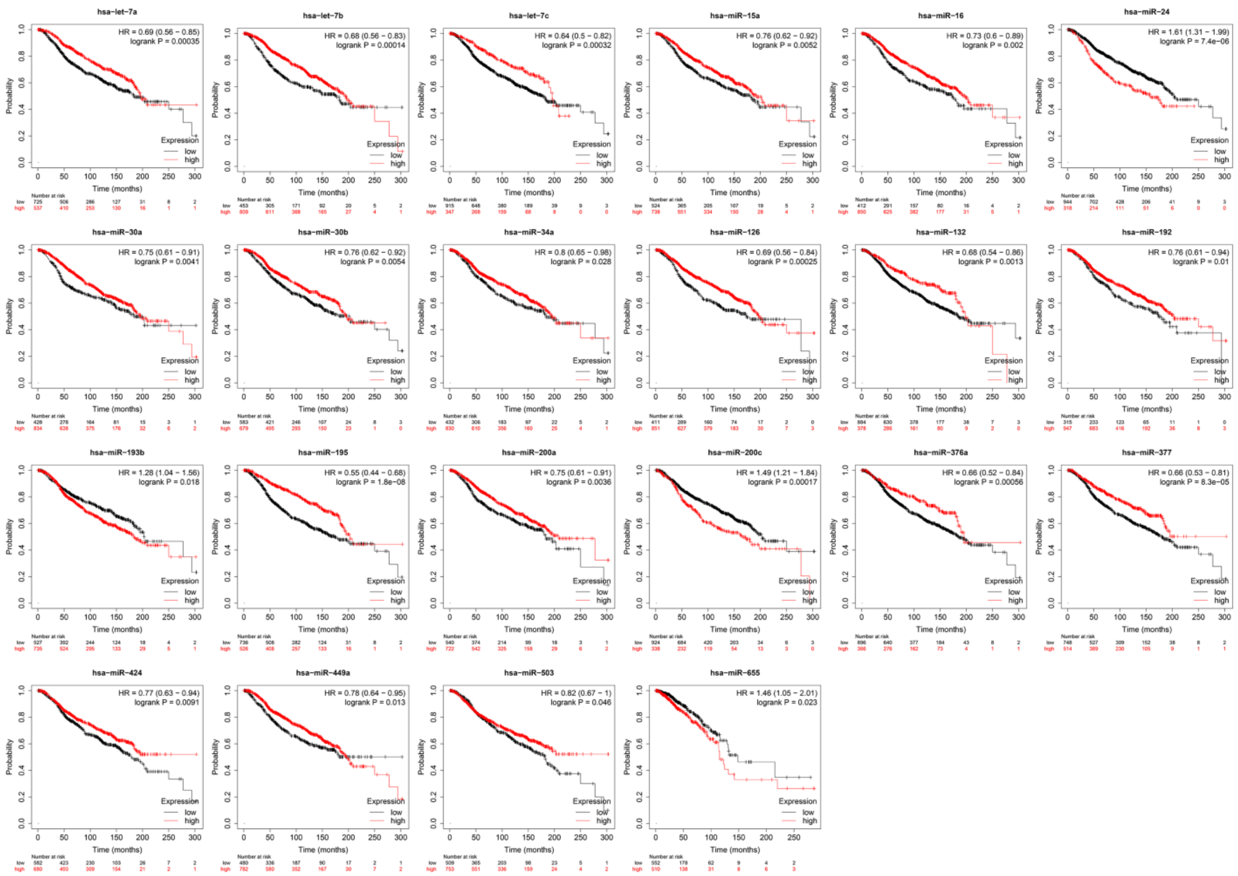

Figure S5 The 34 predicted miRNAs were imported to Kaplan-Meier plotter online tools and 22 out of 34 showed significantly related to worse prognosis in BC patients ($p < 0.05$). Red lines represent high expression genes and black lines means low expression genes in BC patients.

Supplementary Tables

Table S1. The primers sequences for each gene

ID	Sequence(5'- 3')	Product Length(bp)
GAPDH.F	TGTTCGTCATGGGTGTGAAC	154
GAPDH.R	ATGGCATGGACTGTGGTCAT	
CCNB1.F	AATAAGGCGAAGATCAACATGGC	111
CCNB1.R	TTTGTTACCAATGTCCCCAAGAG	
CCNB2.F	CCGACGGTGTCCAGTGATTT	180
CCNB2.R	TGTTGTTTTGGTGGGTTGAACT	
CCNE2.F	GGAACCACAGATGAGGTCCAT	237
CCNE2.R	CCATCAGTGACGTAAGCAAACT	
CDC20.F	GACCACTCCTAGCAAACCTGG	118
CDC20.R	GGGCGTCTGGCTGTTTTCA	
PTTG1.F	ACCCGTGTGGTTGCTAAGG	90
PTTG1.R	ACGTGGTGTTGAAACTTGAGAT	
BUB1B.F	AAATGACCCTCTGGATGTTTGG	184
BUB1B.R	GCATAAACGCCCTAATTTAAGCC	
TTK.F	GTGGAGCAGTACCACTAGAAATG	146
TTK.R	CCCAAGTGAACCGGAAAATGA	

Table S2. All 124 commonly differentially expressed genes (DEGs) were obtained from the intersection of three profile datasets, containing 65 up-regulated genes and 59 down-regulated genes in the breast cancer tissues compared to normal breast tissues

DEGs	Gene Name
Up-regulated	ANLN ASPM AURKA BGN BIRC5 BUB1B CCNB1 CCNB2 CCNE2 CDC20 CDCA3 CDK1 CEACAM6 CENPF CENPU CEP55 CKS2 COL10A1 COL11A1 COL1A1 COL1A2 COMP CTHRC1 CXCL10 DLGAP5 DTL FAM83D FN1 GJB2 GPRC5A HMMR HN1 IFI6 INHBA IQGAP3 ISG15 KIAA0101 KIF18B KIF20A KIF2C LRRC15 MELK MMP1 MMP11 NEK2 NUF2 NUSAP1 PRC1 PRR11 PTTG1 RRM2 S100P SLC35F6///CENPA SQLE TK1 TOP2A TPX2 TRIM59///IFT80 TTK TYMS UBE2C UBE2T UHRF1 WISP1 ZWINT
Down-regulated	ABI3BP ADAMTS5 ANXA1 APCDD1 C2orf40 CA3 CAV1 CAV2 CDKN1C CLIP4 COPG2IT1 CRYAB CXCL14 CXCL2 DMD EFEMP1 EMP1 FAM13A FGF2 FMO2 GGTA1P GNAI1 GNAL GULP1 HLF HOXA10-HOXA9///MIR196B///HOXA9 IRS2 ITIH5 ITM2A LMOD1 LOC100288911 LOC100506558///MATN2 MAGI2-AS3 MAMDC2 MAOA MME MT1M NMT2 NR3C2 NTRK2 PALMD PDGFD PDK4 PLAGL1 PLSCR4 PPP1R14A RBMS3 RNF150 S100B SAA2///SAA1 SAA2-SAA4///SAA2///SAA1 SDPR SFRP1 SORBS1 SYNPO2 TF TGFBR3 TMTC1 TSHZ2

Table S3. KEGG pathway analysis of differentially expressed genes in breast cancer

Pathway ID	Name	Court	*p*-Value	Genes
hsa04110	Cell cycle	8	4.84E-07	CCNE2 CCNB1 CDK1 CCNB2 BUB1B TTK CDC20 PTTG1
hsa04512	ECM-receptor interaction	6	2.47E-05	COMP COL1A2 COL1A1 COL11A1 FN1 HMMR
hsa04114	Oocyte meiosis	6	7.00E-05	CCNE2 CDK1 CCNB2 CDC20 AURKA PTTG1
hsa04115	p53 signaling pathway	5	1.35E-04	CCNE2 CCNB1 CDK1 CCNB2 RRM2
hsa04974	Protein digestion and absorption	4	0.004866151	COL1A2 COL1A1 COL11A1 COL10A1
hsa05146	Amoebiasis	4	0.009271288	COL1A2 COL1A1 COL11A1 FN1
hsa04510	Focal adhesion	5	0.009872682	COMP COL1A2 COL1A1 COL11A1 FN1
hsa04151	PI3K-Akt signaling pathway	6	0.011454163	CCNE2 COMP COL1A2 COL1A1 COL11A1 FN1
hsa05222	Small cell lung cancer	3	0.04593653	CCNE2 CKS2 FN1

hsa04914	Progesterone-mediated oocyte maturation	3	0.048965944	CCNB1 CDK1 CCNB2

Table S4. Validation of 39 genes via GEPIA

Item	Genes
Genes with high expressed in BC (P < 0.05)	ANLN ASPM AURKA BIRC5 BUB1B CCNB1 CCNB2 CCNE2 CDC20 CDCA3 CDK1 CENPF CENPU CEP55 CKS2 DLGAP5 DTL FAM83D HMMR KIAA0101 KIF18B KIF20A KIF2C MELK NEK2 NUF2 NUSAP1 PRC1 PTTG1 RRM2 TK1 TOP2A TPX2 TTK TYMS UBE2C UBE2T UHRF1 ZWINT

Table S5. The prognosis information of the 39 key candidate genes

Category	Genes
Genes with significantly worse survival (P < 0.05)	ANLN ASPM AURKA BIRC5 BUB1B CCNB1 CCNB2 CCNE2 CDC20 CDCA3 CENPF CENPU CEP55 CKS2 DLGAP5 DTL FAM83D HMMR KIAA0101 KIF18B KIF20A KIF2C MELK NEK2 NUSAP1 PRC1 PTTG1 RRM2 TK1 TOP2A TPX2 TTK TYMS UBE2C UBE2T ZWINT
Genes without significantly worse survival (P > 0.05)	CDK1 NUF2 UHRFI

Table S6. Re-analysis of 36 selected genes via KEGG pathway enrichment

Pathway ID	Name	Court	*p*-Value	Genes
hsa04110	Cell cycle	7	1.42E-07	CCNE2 CCNB1 CCNB2 BUB1B TTK CDC20 PTTG1
hsa04114	Oocyte meiosis	5	7.39E-05	CCNE2 CCNB2 CDC20 AURKA PTTG1
hsa04115	p53 signaling pathway	4	3.57E-04	CCNE2 CCNB1 CCNB2 RRM2
hsa00240	Pyrimidine metabolism	3	0.017193361	TYMS RRM2 TK1

Table S7. The predicted interaction of TF-miRNA-mRNA

mRNA	miRNA	TF
CCNE2	hsa-miR-15a-5p	AR ARNTL BRCA1 BRD4 CEBPB CTCF E2F1 E2F4 ERG ESR1 FOXA1 FOXM1 HIF1A KDM5B MYC NRF1 STAT3 TFAP2C ZBTB17 ZNF143
	hsa-miR-16-5p	NA*
	hsa-miR-30a-5p	AR E2F1 ESR1 FOSL1 FOXA1 FOXM1 HIF1A JUN PGR STAT3 TEAD4 TP53 WWTR1 YAP1
	hsa-miR-30b-5p	ESR1 FOS FOXA1 GABPA HDAC2 JUN KDM5B MYC NR2F2 NRF1
	hsa-miR-34a-5p	AR ARNTL CTCF ESR1 FOXA1 HIF1A KDM5B MYC NRF1 TP53 TP63 TRIM25 ZNF143
	hsa-miR-126-3p	CTCF ESR1 HIF1A KDM5B NRF1
	hsa-miR-195-5p	AR BRD4 CTCF E2F1 ESR1 HIF1A KDM5B NRF1 RAD21
	hsa-miR-200a-3p	AR ARNTL ESR1 FOXA1 GRHL2 HIF1A KDM5B SRF TFAP2C TP63
	hsa-miR-200c-3p	AHR AR ARNTL CEBPB CTCF E2F1 EGR1 ESR1 FOS FOXA1 GATA3 HIF1A JUN KDM5B MAX MYC NR2F2 NRF1 TAF1 TFAP2A TFAP2C XBP1 ZNF143
	hsa-miR-424-5p	E2F1 E2F4 ERG KDM5B SFPQ TRIM25 ZBTB17 ZNF143
	hsa-miR-449a	ARNTL CTCF E2F1 E2F4 ESR1 FOXA1 HIF1A KDM5B NRF1 RAD21 TRIM25
	hsa-miR-503-5p	E2F1 E2F4 ERG KDM5B SFPQ TRIM25 ZBTB17 ZNF143
CCNB1	hsa-let-7b-5p	AR E2F1 ESR1 FOXA1 HIF1A STAT3 TFAP2C
	hsa-miR-24-3p	NA*
	hsa-miR-132-3p	ARNTL CTCF CTCFL EGR1 GABPA GATA3 HIF1A KDM5B MAX MYC NRF1 RAD21 REST SMC1A TRIM25
	hsa-miR-377-5p	NA*
CCNB2	hsa-let-7a-5p	NA*
	hsa-let-7c-5p	ARNTL CTCF EP300 ESR1 FOS FOXA1 FOXM1 GATA3 GRHL2 HIF1A KDM5B KMT2C NR3C1 STAT3 TFAP2C
BUB1B	hsa-miR-192-5p	CTCF CTCFL ESR1 ESRRA HIF1A KDM5B

		NR2F2 RAD21 SMC1A
	hsa-miR-193b-3p	ARNTL CTCF E2F1 ERG ESR1 FOS HIF1A KDM5B MYC RAD21 SMC1A ZNF143
CDC20	hsa-miR-16-5p	NA*
	hsa-miR-30a-5p	AR E2F1 ESR1 FOSL1 FOXA1 FOXM1 HIF1A JUN PGR STAT3 TEAD4 TP53 WWTR1 YAP1
	hsa-miR-34a-5p	AR ARNTL CTCF ESR1 FOXA1 HIF1A KDM5B MYC NRF1 TP53 TP63 TRIM25 ZNF143
	hsa-miR-192-5p	CTCF CTCFL ESR1 ESRRA HIF1A KDM5B NR2F2 RAD21 SMC1A
	hsa-miR-193b-3p	ARNTL CTCF E2F1 ERG ESR1 FOS HIF1A KDM5B MYC RAD21 SMC1A ZNF143
TTK	hsa-miR-192-5p	CTCF CTCFL ESR1 ESRRA HIF1A KDM5B NR2F2 RAD21 SMC1A
	hsa-miR-376a-3p	NA*
PTTG1	hsa-let-7b-5p	AR E2F1 ESR1 FOXA1 HIF1A STAT3 TFAP2C
	hsa-miR-655-3p	AR EP300 ESR1 FOXA1 PGR

*NA means no available, because of the low evidence level or without experimental support.
